# Understanding Protein Mobility in Bacteria by Tracking Single Molecules

**DOI:** 10.1016/j.jmb.2018.05.002

**Published:** 2018-10-26

**Authors:** Achillefs N. Kapanidis, Stephan Uphoff, Mathew Stracy

**Affiliations:** 1 Gene Machines Group, Biological Physics Research Unit, Clarendon Laboratory, Department of Physics, University of Oxford, Oxford, OX1 3PU, United Kingdom; 2 Department of Biochemistry, University of Oxford, Oxford, OX1 3QU, United Kingdom

**Keywords:** protein diffusion, single-molecule tracking, anomalous diffusion, macromolecular crowding, target search, GFP, green fluorescent protein, FRAP, fluorescence recovery after photobleaching, PSF, point-spread-function, MSD, mean-square displacements, PALM, photoactivated localization microscopy

## Abstract

Protein diffusion is crucial for understanding the formation of protein complexes *in vivo* and has been the subject of many fluorescence microscopy studies in cells; however, such microscopy efforts are often limited by low sensitivity and resolution. During the past decade, these limitations have been addressed by new super-resolution imaging methods, most of which rely on single-particle tracking and single-molecule detection; these methods are revolutionizing our understanding of molecular diffusion inside bacterial cells by directly visualizing the motion of proteins and the effects of the local and global environment on diffusion. Here we review key methods that made such experiments possible, with particular emphasis on versions of single-molecule tracking based on photo-activated fluorescent proteins. We also discuss studies that provide estimates of the time a diffusing protein takes to locate a target site, as well as studies that examined the stoichiometries of diffusing species, the effect of stable and weak interactions on diffusion, and the constraints of large macromolecular structures on the ability of proteins and their complexes to access the entire cytoplasm.

## Graphical abstract

**Figure:**
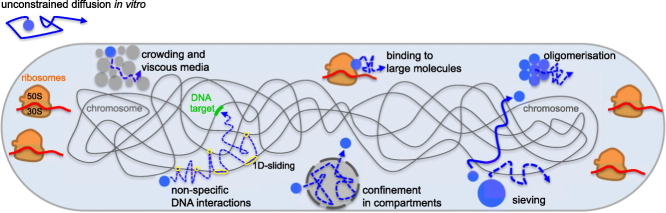

## Introduction

Proteins are elemental structural and functional entities vital for the survival and growth of all biological cells, and their interactions result in a remarkable variety of protein complexes that serve as the molecular machinery, structural frameworks and regulatory switches of each cell. Due to their importance, protein interactions have been studied extensively physically and biochemically *in vitro* using purified systems; however, most of these studies cannot replicate the complexity encountered by proteins in their cellular environment, even for deceivingly “simple” organisms such as *Escherichia coli*. For example, it is extremely difficult to reconstruct *in vitro* the dependence of protein interactions on macromolecular crowding [1], caused by the millions of copies of different proteins, the tens of thousands of ribosomes, and the 4.6-Mbp-long bacterial chromosome. Apart from the large excluded-volume effects (due to large part of the cellular volume being occupied by macromolecules and thus inaccessible to the protein of interest) [2], the abundance of other cellular macromolecules introduce many competing specific and non-specific interactions that can affect substantially the physical behavior and biological function of a protein of interest.

As a result, there have been significant efforts to study protein interactions and their determinants in the natural context of living cells, both for eukaryotes and for prokaryotes. In the case of bacteria, the modest size of bacterial cells (with a characteristic length scale of 1 μm) allows most proteins to move within seconds from one end of the cell to the other simply by harnessing thermal energy (through collisions with the solvent molecules) and without ATP consumption or molecular motors; as a result, bacteria typically do not rely on active machinery for intracellular cargo transport or cytoplasmic mixing. Furthermore, the rate of protein encounters, which controls the kinetics of protein complex formation and subsequent reactions, depends heavily on the cytoplasmic diffusion of each protein and its interacting partners (e.g., another copy of the same protein, a different protein, a site on DNA/RNA or an extra-cytoplasmic component). Diffusion can be rate-limiting for relatively fast processes *in vivo*, as suggested for bacterial chemotaxis [3]. Furthermore, although diffusion can be much faster than subsequent protein-driven processes (e.g., catalytic-driven processes that are completed on the 1-min timescale, such as transcription and translation), some of these processes can be delayed due to global cellular changes that increase crowding and create barriers that slow down or heavily confine diffusion (e.g., during osmotic upshift [4]). Diffusion of proteins, such as transcription factors, has also been suggested to enhance dramatically gene-expression noise [5].

Since protein diffusion is crucial for understanding the formation of protein complexes *in vivo* and has regulatory importance, it has been the subject of many *in vivo* single-cell fluorescence microscopy studies, which, in contrast to studies in fixed cells or *in vitro* reconstituted machineries, can observe the real-time dynamics of diffusion in the natural context. However, *in vivo* diffusion studies have been challenging due to the small size of bacterial cells (e.g., 3 μm long and 0.8 μm wide for a typical *E. coli* cell) and the difficulty in data interpretation due to the ensemble averaging inherent in the initial methods used. In the past 10 years, these limitations have been addressed by the introduction of sensitive, specific and direct optical-microscopy methods for diffusion analysis at the single-molecule level in living bacteria [6], [7], which have provided unique insight about how cellular contents and structures affect diffusion. These methods are also helping the construction of more realistic and quantitative systems-biology models for the kinetics of biological processes *in vivo*.

Here, we review the contributions of single-molecule fluorescence approaches toward understanding how diffusion in living bacteria limits and controls protein interactions, how the cytoplasm acts as a medium for diffusion, and how cellular components affect the kinetics of target location and complex formation via non-specific interactions and confinement effects. The review will discuss mainly intracellular diffusion in the cytoplasm and will not cover diffusion within membranes or the periplasm. We will also briefly discuss the study of cytoplasmic diffusion in single bacteria using ensemble methods; see Ref. [8] for an excellent review on the topic. Readers will also benefit from several insightful reviews on the related topic of macromolecular crowding [9], [10], [11], [12], [13].

## Cytoplasmic diffusion inside bacteria

Diffusion within single bacteria was initially analyzed by quantifying the kinetics of changes of fluorescence signals generated typically by green fluorescent protein (GFP) and its derivatives. GFP has served as an excellent inert tracer for diffusion studies since it has no known specific interactions with the contents of the bacterial cytoplasm; furthermore, GFP tagging offers a simple genetic method for studying the diffusion of proteins of different sizes.

The main microscopy method for studying diffusion was fluorescence recovery after photobleaching (FRAP [14]), which examines the kinetics of protein diffusion by bleaching a small region inside a single cell using a high-intensity laser pulse, and monitoring the return of the fluorescence intensity to a steady-state level; the kinetics can be fit to models that describe the rate of replenishment of fluorescent proteins into the bleached area. Rapid fluorescence recovery shows high protein mobility, whereas incomplete or slow recovery implies the presence of bound molecules either inside or outside the bleached region. The analysis can identify whether an ensemble of labeled molecules moves in the cell primarily through Brownian motion (“free” or “normal” diffusion) or in a non-Brownian fashion (“anomalous” diffusion), which can result from confined diffusion (diffusion within a compartment), diffusion limited by the presence of barriers or molecular interactions, directed motion (“super-diffusion,” e.g., due to active molecular transport) or combinations thereof. The analysis can also capture the approximate fraction of molecules that are immobile.

The first FRAP study of GFP mobility in the bacterial cytoplasm performed by Elowitz *et al*. [15] showed that GFP has an apparent diffusion coefficient D of ~ 7.7 μm^2^/s, a value ~ 11-fold lower that in aqueous solution *in vitro* (87 μm^2^/s; Ref. [16]), and ~ 3-fold lower than GFP diffusion in eukaryotic cells [16]. These differences were attributed mainly to the high levels of macromolecular crowding in bacteria, which result from the extremely high protein concentrations in the cytoplasm [200–300 g/L in *E. coli* (Ref. [2]) and up to 400 g/L in osmotic upshift conditions due to water loss from the cells], as opposed to the very dilute solutions used *in vitro*; the presence of large macromolecules, due to steric hindrance, will shorten the protein displacements that occur as a result of the protein-solvent collisions, effectively slowing down diffusion in a size-dependent manner [17]. This effect is in addition to the decreased diffusion due to the higher viscosity encountered in different regions of the cytoplasm [18]. The pioneering study by Elowitz *et* *al*. also established that one cannot simply attach a single “effective” viscosity to the cytoplasm, since this apparent viscosity will vary for different proteins according to their size and interactions.

Diffusion studies of GFP and GFP-labeled proteins were also performed in different environments in *E. coli* [19], as well as in osmotically shocked bacteria (treated with high concentration of NaCl or sorbitol); the latter study showed that diffusion decreases by ~ 8-fold in moderate osmolarity solutions [20], whereas at high osmolarity, the cytoplasm becomes compartmentalized, with GFP diffusing slowly within individual compartments [20].

Further studies examined the size dependence of protein diffusion in the cytoplasm and suggested the presence of obstacles that act as a “molecular sieve” for larger proteins [21]. A related study of *E. coli* under osmotic stress [4], which used the small fluorescent tracer NBD-glucose, also suggested that high osmolarities cause molecular sieving driven by a condensed nucleoid and increased crowding; in this case, small molecules diffuse unabated, while larger ones (e.g., GFP) slow down substantially. A separate study [22] on covalently linked GFP multimers showed that the diffusion size dependence for multi-GFP protein constructs (with 2–4 GFP units) followed simple diffusion theory (i.e., following the Stokes–Einstein equation, which predicts diffusion coefficients based on the diameter of diffusing molecules in dilute solutions). However, a comprehensive comparison of *in vivo* diffusion coefficients for many proteins of different sizes showed that diffusion slows down more significantly as a function of size than predicted by theory [8].

Early studies [21] also showed that different DNA-binding proteins interact to a different degree with the chromosome, with the nucleoid-associated protein H-NS showing much faster diffusion compared to transcriptional repressor TetR, which binds more stably to specific DNA sites [21]. Other important interactions involve transient binding to other diffusing (and occasionally large) molecules, such as ribosomes, which were shown to slow down diffusion of positively charged proteins, such as GFP derivatives engineered to carry an artificially large positive charge [23].

**Fig. 1:**
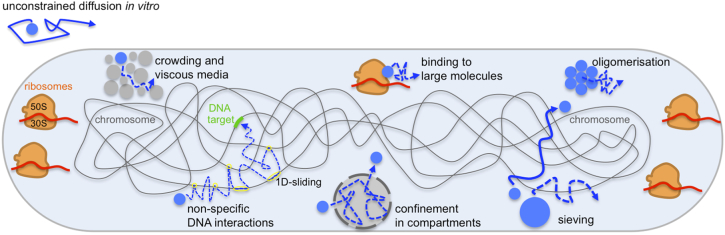
Diffusion modes in the bacterial cytoplasm. Small proteins perform unconstrained diffusion *in vitro.* However, even small proteins such as GFP diffuse 10-fold slower *in vivo* due to macromolecular crowding and increased viscosity. Binding of proteins to large partners (either stable or transient) and oligomerization will also slow diffusion. Non-specific and near-specific interactions can further decrease the apparent mobility of DNA-binding proteins; the same is true for diffusion within transient compartments or regions of confinement. Diffusion can also be affected in a size-dependent way via molecular sieving, for example, through the nucleoid, which can act as a matrix that excludes large macromolecules from its interior.

The FRAP-based studies provided an excellent platform for understanding protein diffusion in the cytoplasm and for dissecting the effects of crowding, confinement, molecular sieving and transient interactions (Fig. 1). However, FRAP studies are inevitably limited by ensemble averaging: as such, they cannot reliably describe complex diffusion landscapes, such as mixed populations, molecules that interconvert between different modes of diffusion and molecules that show sub-diffusion. FRAP is also technically difficult to implement in small cells such as bacteria, since the focused bleaching beam has dimensions (~ 300 nm laterally, ~ 500 nm axially) close to the scale of bacterial cells [20]. To circumvent this issue and counteract the effects of cellular confinement on diffusion, many FRAP studies relied on imaging artificially elongated bacteria [21], generated after treatment with the antibiotic cephalexin, which blocks cell wall growth and cell division. However, as a bacteriostatic drug, this treatment can strongly affect cell behavior so it is important to verify that the observations in cephalexin-treated cells also apply to normal growth conditions. For example, the regulation of the general stress response in *Bacillus subtilis* and the adaptive response in *E. coli* was significantly altered by cephalexin treatment, because the increased cell volume reduced the effects of molecular noise [24], [25].

## Studying molecular diffusion via single-particle tracking

The landscape for characterizing protein diffusion in live bacteria changed significantly upon the introduction of single-particle tracking [26], which addressed the limitation of ensemble averaging and provided direct access to the motions and interactions of single protein molecules in the cytoplasm. The first tracking studies of diffusion in the bacterial cytoplasm used large particles labeled with multiple fluorophores [27], [28]; this labeling approach (based on the high-affinity interaction between an RNA hairpin and a GFP fusion of a capsid protein from bacteriophage MS2) provided strong fluorescence signals to facilitate tracking *in vivo* for extended periods.

Soon after, the ability to detect single proteins inside living bacteria was established and was used to study gene expression heterogeneity in bacteria by counting expressed proteins that become anchored on the membrane [29]. For an extensive discussion of the challenges that must be overcome in order to achieve *in vivo* single-molecule detection, and the principles that allowed its implementation, see a number of reviews [6], [7], [30].

The ability to detect single proteins *in vivo* enabled single-molecule tracking, that is, applying the principles of single-particle tracking to single fluorescent proteins. Bacteria are well suited for single-molecule tracking because cytoplasmic molecules remain within a single focal plane [31]. The method uses wide-field imaging to track the location of a molecule over time and to generate spatial trajectories. The location of each molecule is recovered using high-precision localization [32], which identifies the position of a single fluorescent molecule from its image; if the molecule is immobile, its image will match the point-spread-function (PSF) of the microscope, that is, the diffraction-limited 2-D image of a single fluorophore, approximated well by a 2-D Gaussian distribution with a width of ~ 250 nm; the centroid of this Gaussian will reflect the position of the molecule.

**Fig. 2:**
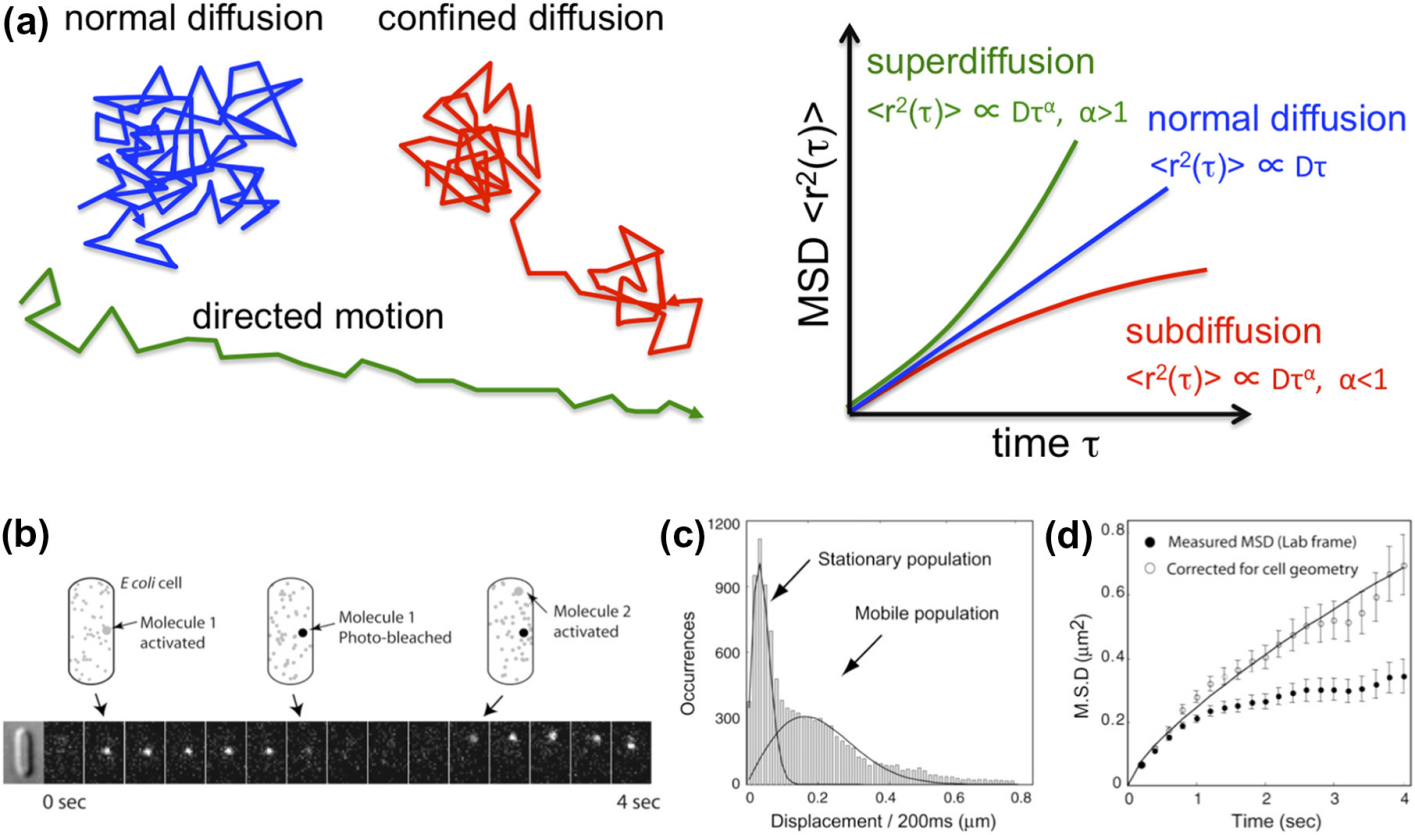
Molecular diffusion and MSD analysis. (a) Different types of diffusion will result in different molecular tracks (left panel), which in turn will lead to a different shape for the MSD plot versus time (right panel). The plot can indicate the spatial and temporal scales for the factors that affect diffusion, for example, by identifying the mean size of the container that causes confinement and the timescale during which the confinement effects are manifested. (b–d) Photoactivated PALM microscopy: first application in living bacteria. Example of sparse activation of Dendra2-FtsZ molecules (panel b), followed by an analysis of molecular mobility using single-step displacements (panel c), and an MSD analysis for the mobile population, which is shown to perform anomalous diffusion (panel d). Panels b–d are adapted from Ref. [33].

Using the trajectories (or “tracks”), one can identify whether molecules move in a Brownian or non-Brownian fashion (see FRAP section). Identifying the diffusion mode can be done using analysis of mean-square displacements (MSD), which recover properties such as diffusion coefficients, confinement or clustering area, velocity of directed motion, and anomalous diffusion exponents. To generate an MSD plot, a single trajectory is analyzed in terms of the 2-D displacement of a molecule for different time lags (Fig. 2). The MSDs for free 2-D diffusion scale linearly with lag time (MSD = 4 Dt), with the slope providing an estimate of diffusion coefficient *D*. The MSD dependence on time is more complex for non-Brownian diffusion, with anomalous diffusion following a relation of MSD = 4 Dt^α^, where an exponent of *α* < 1 indicates subdiffusion due to steric barriers, crowding or binding to cellular structures. The smaller the value of *α*, the greater the deviation from Brownian diffusion; for example, large RNA-protein complexes with ~ 100-nm diameter showed sub-diffusive behavior with *α* = 0.70 in the bacterial cytoplasm [28]. The value of *α* can also be > 1, indicating super-diffusion, for example, due to directed motion.

The first example of tracking single-protein motion in bacteria addressed the question of how transcription factors locate their targets in cells [34]. Transcription factors control much of gene expression in cells and can locate a site on a chromosomal DNA fragment *in vitro* with rates up to 100-fold faster than expected on the basis of pure 3-D diffusion. The study used an inventive stroboscopic approach that illuminated a YFP fusion of the *lac* repressor (LacI) for a short time during which minimal protein movement occurs, even when a protein diffuses freely in the cytoplasm. Equipped with this technique and MSD analysis, Elf *et al.* studied both the specific and non-specific interaction modes of LacI with DNA, characterizing the 3-D diffusion of LacI in the cytoplasm and showing that the protein spends ~ 90% of its time performing 1-D diffusion on DNA (while dissociating from DNA within 5 ms). These findings provided strong support for a combined 1-D and 3-D diffusional search mode for target search, and highlighted the importance of non-specific interactions for protein mobility *in vivo*.

## Tracking of single photoactivatable proteins

Tracking using fully fluorescent proteins was restricted to low densities of fluorescent molecules at any given time (1–10 molecules per cell), since it is otherwise difficult to detect individual molecules due to the overlap between their images. For example, if one considers the size of a typical bacterial cell (800-nm diameter and 3 μm in length), the size of a PSF (250 nm wide), and the fact that the fluorophore image may be wider than the PSF (due to diffusion within the exposure time), having even 10 fluorescent molecules in a single cell leads to a crowded situation that makes single-molecule tracking inaccurate or impossible. As a result, it is difficult to study the behavior of most proteins in living cells at the native state or copy numbers using fully fluorescent protein fusions. Furthermore, it is not possible to generate large statistics from an individual cell, thus missing the opportunity to study cell-to-cell heterogeneities that may reflect molecular subpopulations due to chemical heterogeneity (covalent or non-covalent) or different cellular environments.

These limitations were overcome by combining single-particle tracking with photoactivation, a process central to photoactivated localization microscopy (PALM [35]). The combined method, termed spt-PALM, or simply “tracking-PALM,” was first demonstrated in studies of protein diffusion and clustering on membranes of mammalian cells [36], shortly followed by a similar method applied to the tubulin-homologue FtsZ in *E. coli* [33] (Fig. 2c–e), followed by further extensions [37], [38].

In tracking-PALM, a protein of interest is fused to a photoactivatable fluorescent protein, such as Dendra2 [39] or PAmCherry [40]. As in PALM studies, single molecules are photoactivated using 405-nm (or near-UV) illumination, imaged upon excitation by a different laser, and then photobleached irreversibly (Fig. 2b). To ensure the presence of very few emitting (photoactivated) molecules at any given time, the power of the activating laser is adjusted to achieve sparse photoactivation which, in combination with the excitation-laser power, ensures that each cell contains at most one molecule for most movie frames; this facilitates linking localizations to form tracks, especially for highly mobile molecules that move significantly between frames. The excitation laser intensity is also adjusted to detect enough photons for a high localization precision per molecule, and to collect enough frames prior to photobleaching (to minimize the statistical error in calculating diffusion coefficients from single-molecule tracks). This fine balance is key for the generality of tracking-PALM, which can then address proteins of *any* copy number.

**Fig. 3:**
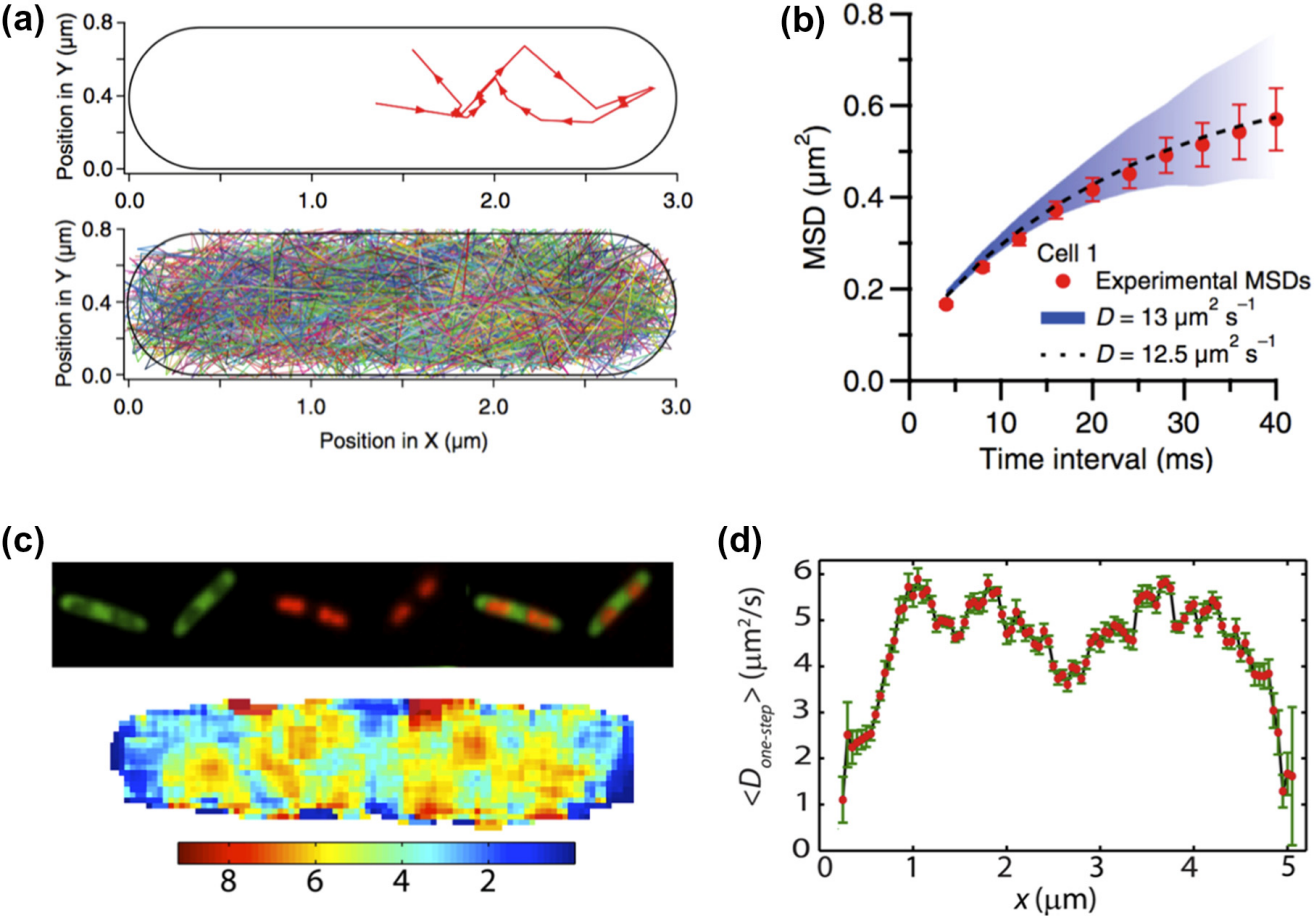
Using small photoactivatable proteins as tracers of cytoplasmic diffusion. (a and b) Use of mEos2 tracking via stroboscopic imaging in the *E. coli* cytoplasm. Numerous single tracks are generated per cell, and they are used to look for the spatial distribution of mEos2 (panel a), as well as its overall diffusion behavior using MSD analysis (panel b). Panels a and B are adapted from Ref. [37]. (c and d) Use of Kaede tracking via fast imaging in the *E. coli* cytoplasm. The nucleoid and ribosomes show segregation within bacteria, with ribosomes occupying mainly the polar areas and the middle of the cell (panel c, top). The one-step Kaede apparent diffusion coefficient (panel c, bottom) shows heterogeneity that correlates with the ribosome-nucleoid segregation, with the Kaede showing slower diffusion in ribosome-rich areas (panel d). Panels c and d are adapted from Ref. [41].

Once all molecules are localized, tracks are generated and analyzed to calculate an apparent diffusion coefficient for each molecule or to recover the distribution of all displacements (Fig. 2c); this allows sorting of molecules according to mobility. The tracking information also enables MSD analysis to identify modes of diffusion (Fig. 2d). Since the number of tracks can be large, very dense maps of diffusion can be generated per cell (e.g., see Fig. 3a) and used for characterizing cellular microenvironments and molecular subpopulations in a single cell.

Despite the apparent simplicity and direct nature of tracking-PALM measurements, there are many issues to consider while designing experiments and interpreting tracking data [30], [42]. Examples of such complications include the following: the need for cells to be immobilized flat on the agarose surfaces typically used, the need for robust cell segmentation, the presence of incomplete and variable folding of different photoactivated FPs in different cellular contexts, the presence of FP blinking that splits larger tracks to shorter ones, the difficulty in tracking very fast diffusing species quantitatively, and the difficulty in identifying diffusion subpopulations with modest differences in their apparent diffusion profile. Active research in many labs is currently addressing these limitations.

## Protein mobility questions tackled by single-molecule tracking

What aspects of *in vivo* protein diffusion can be tackled by tracking using either photoactivatable or fully fluorescent FPs? Single-molecule tracking offers additional temporal and spatial resolution without ensemble averaging, as well as high throughput; for example, using photoactivatable FPs as tracers, one can generate large sets of intracellular tracks to examine cytoplasmic diffusion and microviscosity, effects of crowding, confinement and sieving. Diffusion can be studied in different subcellular regions of a single cell, offering a systematic view of non-specific interactions with large structures such as the bacterial chromosome. Furthermore, the high information content of the tracking measurements along with perturbations provided through the use of mutants or chemically treated strains can help us determine how and to what extent cytoplasmic diffusion is affected by purely “steric” effects (physical confinement by compartment, walls and barriers) and weak chemical interactions (such as electrostatic interactions and van der Waals forces) [13].

One can also estimate the average search time for a protein to find its cellular target. This time involves the entire trajectory from the point of protein dissociation from one specific target to the next target-binding event; the search depends both on the size of diffusing protein and on all its interactions with the diffusion medium and interacting components. Some interactions during the search can be very specific, such as complex formation via binding to another protein partner; the diffusion analysis allows these crucial interactions to be identified and characterized.

## Tracking cytoplasmic diffusion of small proteins and large particles

Not surprisingly, some of the first PALM-based diffusion studies focused on tracking small photoactivatable proteins. Extending the methodology used to study *lac* repressor diffusion [34], English *et al*. [37] used stroboscopic illumination to study the diffusion of mEos2 (a monomeric fluorescence protein of 26 kDa in size). Use of short illumination times (to minimize motion blurring) and 4-ms exposures yielded thousands of tracks per cell and showed that mEos2 explored the entire cell. The fast motion of mEos2 led to deviations from the linear relation between MSD and diffusion time, attributed to cellular confinement (Fig. 3a and b); however, simulations that account for projection and confinement effects showed that mEos2 essentially undergoes Brownian (free) diffusion in cells, without being perturbed by large structures such as the bacterial chromosome.

Similar work was done on Kaede, a photoactivatable protein present primarily as a homotetramer of 116 kDa in cells [41]. As with mEos2, Kaede explored the entire cell and was not excluded by the nucleoid; furthermore, the MSD analysis showed no evidence of subdiffusion for a protein the size of Kaede. There was, however, a small tendency for slower diffusion within the ribosome-rich areas of the cytoplasm, while there was no significant sieving by the nucleoid (Fig. 3c and d). The diffusion coefficient for Kaede was ~ 7.3 μm^2^/s, ~ 1.3 times smaller than that of GFP under similar cellular conditions, and in general agreement with the diffusion slow down expected on the basis of Kaede's size.

The results with proteins of small-to-moderate size (25–100 kDa) can be contrasted with those on a single-particle tracking study in *Caulobacter crescentus* and *E. coli* which examined the intracellular diffusion of large particles such as storage granules, plasmids and self-assembling viral particles [43]. The study showed that, when the cellular metabolism is heavily curtailed or shut down, diffusion of large particles (> 100 nm in diameter) is substantially constrained, with the cytoplasm becoming “glass-like” (i.e., changing from a liquid-like to solid-like phase in a manner that depends on the size of the diffusing species); in contrast, active metabolism made the cytoplasm more “fluid.” Glassy behavior was attributed to crowding effects that result in a 40%–60% excluded volume in *E. coli* [2], with these effects expected to be accentuated by interactions (hydrophobic, electrostatic, etc.) between the macromolecules surrounding the diffusing species. The effect of diffusion slow-down in energy-depleted cells was heavily dependent on the size of the diffusing units, with MSD analysis showing a major effect for > 30-nm particles; in contrast, free GFP diffuses similarly regardless of metabolic status in *C. crescentus* [44]. Intriguingly, the mobility difference between metabolic states was due to large differences in the fraction of two types of particles that exhibit “fast” and “slow” motion; the slow subpopulation was attributed to caging by neighboring macromolecules, which can increase their mobility during active metabolism and thus allow “slow” particles to escape. These results pointed to the presence of “dynamic heterogeneity” within the *same* cell as opposed to the presence of different cellular environments in different cells. How exactly metabolism leads to uncaging of large particles is still an open question.

## Estimating target search times

A straightforward way to measure search times *in vivo* would be to measure the time between target-binding events for single molecules; unfortunately, search times for a single molecule are often on the minute-to-hour timescale, which is currently not easily accessible to continuous tracking of molecules. There are, however, more indirect approaches that rely on protein mobility to estimate search times even on the hour timescale. An excellent example is the study of the target search by the CRISPR–dCas9 complex, a machine that locates a specific sequence within the chromosome via complementary DNA–RNA interactions [45]. The assay relied on measuring the rate required for a labeled dCas9-guideRNA complex to bind to a specific DNA sequence after that DNA target is freed due to the controlled dissociation of a target-occluding LacI molecule. Binding is detected in a single cell when an immobile molecule (imaged using very long exposure times; 5 s) appears at time *t* after removing LacI from the target site by adding IPTG. The study showed that a single dCas9 molecule takes ~ 6 h to find its target, with the search involving a huge number of non-specific binding events to ~ 10^6^ potential targets (which include sequences containing the protospacer adjacent motif), each lasting for < 30 ms. To counter the slow nature of the search of each individual molecule, cells employ several hundred Cas9 molecules and guide RNAs for the target search, so that it takes only ~ 1 min on average for any one of the molecules to find the correct site.

Another way to estimate search times relies on measuring the target-bound fraction of a certain protein and its characteristic binding time. This approach was used to estimate how long DNA polymerase and DNA ligase take to locate a gapped and a nicked DNA site, respectively [38]. Tracking led to diffusion coefficient histograms that provide the bound fraction for each protein. Furthermore, long exposure times coupled with low excitation power (to extend the survival of the fluorophores) and consideration of bleaching rates provided the characteristic bound times for the DNA polymerase and ligase *in vivo* (2.1 s for Pol1 and 2.5 s for Ligase). These times reflect both the reaction time and the time the protein needs to dissociate from the DNA. Using the bound fraction and protein binding time while assuming a homogeneous behavior, one can estimate that in the absence of damage, Pol1 and Lig find their target in ~ 85 s and ~ 63 s, respectively. These times were reduced by ~ 7-fold after treating cells with DNA damage agents, mainly due to the large increase in the number of substrate sites available for binding.

## Uncovering stable interactions and stoichiometry of diffusing species

Diffusion analysis based on single-molecule tracking can also report on the protein binding and interaction stoichiometry of the “searching” unit, which may be a single protein molecule, a stable protein homo-oligomer, a stable complex of proteins or combinations of the above (in case of transient interactions). This ability was used in examining the mobility of a Dendra2-derivative of RelA, a protein central to the stringent response in bacteria [37]; RelA synthesizes the nucleotides pppGpp and ppGpp (collectively known as (p)ppGpp), small molecules that act as “alarm” signals that reprograms gene expression. Under conditions where RelA is expected to be inactive, RelA was largely immobile and matched the diffusion profile of the ribosome, as expected for the association of RelA with the ribosome. In contrast, under induction of amino-acid starvation, RelA diffused much more rapidly, showing a profile similar to free mEos2; these results suggested that RelA, while active, was dissociated from the ribosome, supporting a hopping model for the RelA activity. However, the exact mechanism is still controversial, since a subsequent study [46] using three different RelA fluorescent fusions showed that RelA becomes slow diffusing (with a mobility similar to that of the 70S ribosome) during stringent response, consistent with a model where (p)ppGpp synthesis occurs while RelA is bound to the ribosome; the latter study [46] also underlines how seemingly similar fluorescent proteins may affect the function of the tagged protein of interest in different ways.

**Fig. 4:**
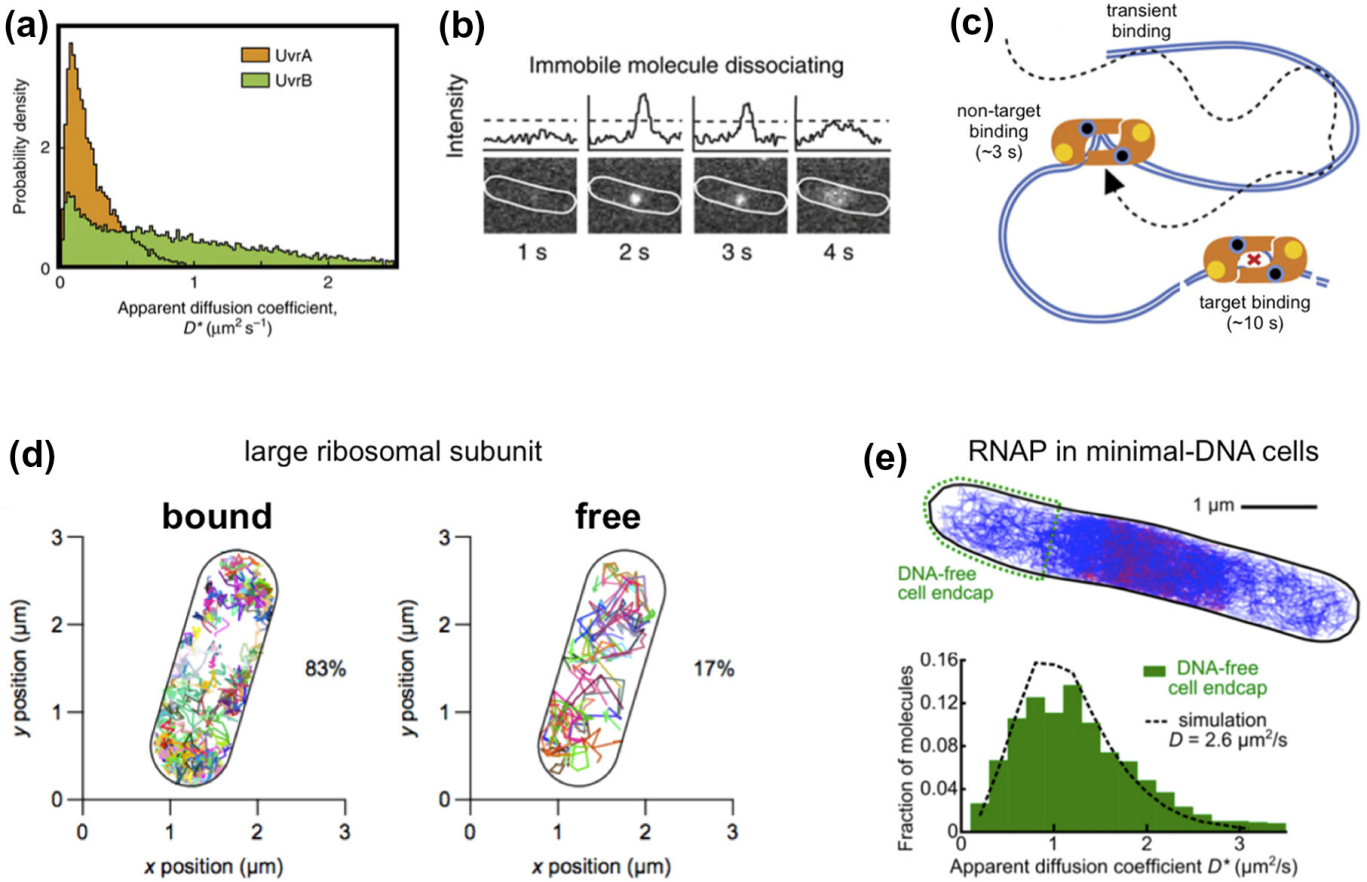
Use of tracking-PALM to characterize the diffusion, interactions and exclusion areas of proteins and their complexes. (a) The apparent diffusion coefficient of UvrA and UvrB are dramatically different, with most UvrA showing stable binding to DNA, and most UvrB showing much faster diffusion. (b) Example of the detection of a full binding event of UvrA to non-target DNA; the blurred image at 4 s indicates protein dissociation from the DNA (as opposed to photobleaching). (c) A schematic summary of the DNA-binding modes of UvrA with the DNA in the presence of bulky DNA damage. Panels a–c are adapted from Ref. [47]. (d) The diffusion profile from single bacterial cells shows that free ribosomal subunit has full access to the nucleoid area (roughly highlights by the tracks on the right panel), where bound subunits are largely excluded from the nucleoid. Adapted from Ref. [48]. (e) The diffusion profile of RNA polymerase in cells with minimal DNA leaves DNA-free regions closed to the cell endcaps (top panel); diffusion from these regions shows that RNA polymerase diffuses much faster than in the presence of DNA and consistent with a corrected diffusion coefficient of 2.6 μm^2^/s, as opposed to 0.4 μm^2^/s in the presence of DNA. Adapted from Ref. [49].

Another tracking study examined the stoichiometry of the DNA-repair machinery responsible for initiating nucleotide excision repair in bacteria [47]. Textbook descriptions of the nucleotide excision repair pathway identify a complex of UvrA and UvrB as the unit that locates and binds to bulky lesions on DNA and initiates DNA repair. However, Stracy *et al*. used comparisons of the diffusion profiles of UvrA and UvrB in cells before and after DNA lesions were induced by exposure to UV light (Fig. 4a; along with controls that involved overexpression of proteins and several mutants) to show that the predominant searching unit is dimeric UvrA alone, which binds to DNA either non-specifically or in a lesion-specific manner (Fig. 4b and c); the latter more stable binding sets the stage for the subsequent association of UvrB molecules that recruit the rest of Uvr proteins to the repair intermediate and complete the repair process.

## Exclusion from subcellular areas

The location of protein tracks relative to different cellular structures can also report on the degree of exclusion of protein complexes from specific regions of the cell. Excellent examples of such studies come from tracking single ribosomes, as in studies of the spatial distribution of the transcription and translation machinery in *E. coli* [31]. Using a YFP version of ribosomal protein S2 (as a proxy for mature 70S ribosome diffusion) and a method of reversible photobleaching to generate single tracks in cells, it was shown that S2 (which is mainly in fully assembled 70S ribosomes and, to a smaller extent, in 30S subunits) mainly diffused within the cell endcaps and the central regions, and was excluded from the nucleoid (that occupies the remaining cellular regions); in contrast, RNA polymerase localized in the nucleoid. Other large structures that are excluded from the nucleoid but diffuse in the nucleoid-free regions include high-copy bacterial plasmids [50].

Further analysis of ribosome mobility based on tracking single ribosomal proteins [48] and examination of the spatial distribution for species of different mobility showed that, although fully assembled ribosomes are excluded from the nucleoid, free ribosomal subunits can access the bulk of the nucleoid without difficulty, and thus have full access to nascent mRNA (Fig. 4d). This picture was further defined by a separate study that examined the diffusion profile of RNA polymerase [49] and used it to identify searching and gene-bound populations of the polymerase. Unclustered polymerases occupied the entire nucleoid, whereas moderately clustered polymerase were preferentially located in the nucleoid periphery; this separation suggested that transcription (and translation) can start anywhere in the nucleoid, but soon after the initial events, the coupled transcription-translation apparatus moves to the nucleoid periphery.

It is also worth stressing that the spatial profile of large machineries may vary substantially in different bacteria, especially given the large structural and functional differences between bacterial species. This was reflected clearly in a recent study [51] that examined the localization and mobility of ribosomes and RNase E (a major protein of the bacterial RNA degradosome) in *C. crescentus*; contrary to *E. coli*, *C. crescentus* contains ribosomes that form weak clusters throughout the cytoplasm and degradosomes that accumulate in clusters along the central cellular axis and away from the cytoplasmic membrane.

## Non-specific interactions

So far we discussed tracking studies of proteins that form stable interactions with other biomolecules and studies of proteins excluded from macromolecular matrices such as the nucleoid. Proteins can also interact non-specifically with long macromolecules and large-scale structures, such as the chromosomal DNA, which serves as the substrate for many reactions; the first tracking work in bacteria [34] had already extensively looked at such interactions, showing that LacI spends much of its search time non-specifically bound to DNA for times shorter than 5 ms. Binding of LacI can also be near specific (i.e., to sequences that are closely related to the cognate sequence, e.g., the *lac* operator in this case). To these interactions, one should also add the presence of chromosomal DNA motions within the nucleoid, which have been suggested to accelerate target search for DNA-binding proteins such as LacI [52].

Interestingly, the Stracy *et al*. work [49] on RNA polymerase also showed that mobile molecules of RNA polymerase spent a significant fraction of their time non-specifically bound to DNA, to the point that it is rare to see excursions of RNA polymerase outside the nucleoid. Experiments with modified cells to engineer DNA-free regions at the polar end-caps of single cells (Fig. 4e) helped to characterize RNA polymerase diffusion in the absence of DNA; the comparison of corrected diffusion coefficients for nucleoid-based and DNA-free protein diffusion revealed that the polymerase spends ~ 85% of its time non-specifically bound to DNA during promoter search—this high value matches the one seen for LacI and exceeds substantially similar estimates for DNA-binding proteins in eukaryotic cells [53] (possibly due to the presence of nucleosomes).

Garza de Leon *et al*. [54] looked at the relative contributions of the specific, near-specific and non-specific binding using tracking-PALM by using strains with overexpressed LacI (i.e., showing mainly non-specific binding), as well as strains containing a chromosome devoid of any *lac* operators (i.e., showing near-specific and non-specific binding) and strains containing a LacI without a DNA binding domain (i.e., incapable of any DNA binding).

The emerging picture for DNA-binding proteins, further supported by studies of the diffusion of numerous DNA-binding proteins in the cytoplasm of bacteria where all chromosomal DNA had been degraded (M.S., S.U., C. Lesterlin, D. Sherratt, A.N.K., in preparation), is that they interact intimately with the chromosomal DNA in the nucleoid. These interactions facilitate target searches using a combination of non-specific binding (with or without 1D sliding) and 3D diffusion. This is also the case for protein machinery assisted by nucleic acids in the process, as the example of CRISPR–dCas9/guide RNA complex with its combined cognate and non-cognate sequence searching mode [45].

## Emerging themes, open questions and future directions

The examples provided offer a taste of how the complexities of protein diffusion in bacteria can be disentangled using powerful single-molecule tracking methods. Even with a limited number of examples, some general themes emerge regarding the factors that affect protein mobility *in vivo*.

The time to locate a target can be very long, and it is often the increase in the copy number (along with the energetic cost associated with making the necessary transcripts and proteins) that compensates for the slow process of target search. Furthermore, the role of non-specific interactions with the chromosome as a substrate is a crucial element for target location and for enhancing the association of the searching protein for the target of interest. So far, the DNA-binding proteins examined using molecular tracking have been seen to spend a high fraction of their time non-specifically bound to DNA.

Regarding the role of the nucleoid as a sieve, the emerging consensus is that its effective pore size is large enough to allow diffusion of small to fairly large proteins (e.g., RNA polymerase, a ~ 450-kDa protein of ~ 20 nm in diameter, or free ribosome subunits, with a similar size), but small enough to exclude larger complexes such as (poly)ribosomes fully assembled on mRNA (with diameter of > 50nm) [55]. This nucleoid permeability and the general diffusion landscape in bacteria change dramatically when cells enter specific physiological states that increase the excluded volume or change cell composition; diffusion then becomes highly complex in terms of the molecular interactions with the microenvironment, which can also lead to confinement of molecules in compartments.

There are also many unresolved questions; how do molecules diffuse in heterogeneously distributed matrices such as the nucleoid, and can we correlate the dynamic heterogeneity of the matrix with the mobility of the diffusing unit? What is the physical basis of a “glassy” cytoplasm and what exactly controls its fluidity? How different is the 1D diffusion mode for different DNA-binding proteins? How important are protein hydrodynamics [56] for *in vivo* diffusion? How different is protein diffusion in bacteria other than *E. coli*, and in more complex organisms?

Some of these questions will require better experimental and computational tools. Some of these tools will rely on increasing the information content from the available photons and thus increasing either the resolution or observation span of the tracking measurements. Such improvements are achieved by a new super-resolution method, MINFLUX [57], which combines photo-activation and complex illumination patterns to localize molecules with precision down to 1 nm, and track large assemblies with 100-fold better temporal resolution. Other improvements should address the limitation of most tracking studies to just two dimensions, which can complicate analysis of diffusion dynamics, especially for membrane-related diffusion; this limitation is in part due to the fact that the popular astigmatism-based method for 3D localization and tracking is not well suited for tracking due to motion blurring. Approaches for 3D tracking based on multi-focus microscopy [58] and double-helix PSFs [59] can help address such technical challenges and provide more direct information regarding 3D diffusion in bacteria.

Furthermore, the use of brighter fluorophores will allow studies with high spatio-temporal resolution or observation span. Such studies include the use of the SNAP/Halo-tag labeling [60], [61], where cell-permeable fluorophores are introduced in cells and bind to a SNAP/Halo-tag fusion of the protein of interest (see Ref. [62] for an example). An alternative approach uses the introduction of labeled proteins or protein substrates in cells via electroporation [63]; this approach is fully compatible with the use of doubly labeled proteins and single-molecule FRET signals, and therefore can allow direct monitoring of the conformations of protein molecules as they diffuse and search, and as they non-specifically or transiently interact with their targets.

The use of brighter fluorophores will also allow faster tracking and observation of rapid changes between different diffusive states; this effort will be substantially aided by computational approaches based on hidden Markov modeling (see Ref. [64]) or kinetic modeling [65]. These approaches will work well with computational models of diffusion that entertain more complex scenarios of protein diffusion *in vivo*, and differentiate between different mechanisms that slow down diffusion. At the end, the combination of the powerful tools should allow bacteriologists and biophysicists to reach a deep understanding of diffusion in bacteria, and to help us appreciate the versatility and ingenuity of these tiny but endlessly fascinating microorganisms.
